# 

**Published:** 1873-11-15

**Authors:** 


Renew Your Subscription.—With the close of the present volume, next month, many of our subscriptions will terminate. We would ask subscribers to renew promptly, so as to avoid trouble and delay in receiving the numbers for next year. We shall adhere *strictly* to the system of advance payment and shall stop the numbers promptly with the termination of subscription unless renewal is made. The amounts are small—but a trifle to each one—but amounting in the aggregate to a considerable sum; the prompt receipt of which is necessary for the proper support and carrying out of our enterprise. Our aim in the future will be, as it always has been in the past, to deserve the patronage and support of the profession by constantly maintaining the value and efficiency of our journal.

We have effected arrangements so that we can offer THE EXAMINER in club with the New York *Medical Record* at $6.00, and with the Philadelphia *Medical and Surgical Reporter* at $7.00. Any other of our home or foreign journals can be had with THE EXAMINER at a discount of from ten to fifteen per cent. on the gross amount of subscription. Any new subscribers, or old subscribers renewing their subscriptions, can also have a copy of Davis' Clinical Lectures with THE EXAMINER at $5.00.

